# Patient and Stakeholder Engagement in the PCORI Pilot Projects: Description and Lessons Learned

**DOI:** 10.1007/s11606-015-3450-z

**Published:** 2015-07-10

**Authors:** Laura P. Forsythe, Lauren E. Ellis, Lauren Edmundson, Raj Sabharwal, Alison Rein, Kristen Konopka, Lori Frank

**Affiliations:** Patient-Centered Outcomes Research Institute (PCORI), 1919 M St NW, suite 250, Washington, DC 20036 USA; AcademyHealth, Washington, DC USA

**Keywords:** patient-centered outcomes research, comparative effectiveness research (CER), patient engagement

## Abstract

**BACKGROUND:**

Patients and healthcare stakeholders are increasingly becoming engaged in the planning and conduct of biomedical research. However, limited research characterizes this process or its impact.

**OBJECTIVE:**

We aimed to characterize patient and stakeholder engagement in the 50 Pilot Projects funded by the Patient-Centered Outcomes Research Institute (PCORI), and identify early contributions and lessons learned.

**DESIGN:**

A self-report instrument was completed by researchers between 6 and 12 months following project initiation.

**PARTICIPANTS:**

Forty-seven principal investigators or their designees (94 % response rate) participated in the study.

MAIN MEASURES

Self-report of types of stakeholders engaged, stages and levels of engagement, facilitators and barriers to engagement, lessons learned, and contributions from engagement were measured.

**KEY RESULTS:**

Most (83 %) reported engaging more than one stakeholder in their project. Among those, the most commonly reported groups were patients (90 %), clinicians (87 %), health system representatives (44 %), caregivers (41 %), and advocacy organizations (41 %). Stakeholders were commonly involved in topic solicitation, question development, study design, and data collection. Many projects engaged stakeholders in data analysis, results interpretation, and dissemination. Commonly reported contributions included changes to project methods, outcomes or goals; improvement of measurement tools; and interpretation of qualitative data. Investigators often identified communication and shared leadership strategies as “critically important” facilitators (53 and 44 % respectively); lack of stakeholder time was the most commonly reported challenge (46 %). Most challenges were only partially resolved. Early lessons learned included the importance of continuous and genuine partnerships, strategic selection of stakeholders, and accommodation of stakeholders’ practical needs.

**CONCLUSIONS:**

PCORI Pilot Projects investigators report engaging a variety of stakeholders across many stages of research, with specific changes to their research attributed to engagement. This study identifies early lessons and barriers that should be addressed to facilitate engagement. While this research suggests potential impact of stakeholder engagement, systematic characterization and evaluation of engagement at multiple stages of research is needed to build the evidence base.

**Electronic supplementary material:**

The online version of this article (doi:10.1007/s11606-015-3450-z) contains supplementary material, which is available to authorized users.

## INTRODUCTION

Patients and other key healthcare stakeholders are increasingly becoming engaged in the planning and conduct of biomedical research.^1^,^2^ Patients and stakeholders can be engaged across stages of research including identifying study topics, choosing hypotheses, analyzing data, and disseminating findings^3^–^5^ (see Table 1 for illustrative examples). Levels of engagement range from consultation, to collaboration in bi-directional partnerships with researchers, to stakeholder-directed projects.^6^ Participatory research approaches have been increasingly recognized as potentially beneficial in several countries,^7^–^10^ and a robust community-based participatory research literature documents key issues in including communities in research.^11^ In the US, growing support for engaged health research is demonstrated by patient engagement programs at several federal health agencies.^12^–^15^ In 2010, the Patient-Centered Outcomes Research Institute (PCORI) was established to fund comparative clinical effectiveness research (CER) to assist patients, clinicians, and other healthcare stakeholders in making informed health decisions.^16^ PCORI requires research engagement in all its funded CER studies.^17^

**Table 1.** Illustrative Examples from the Peer-Reviewed Literature of Patient and Stakeholder Activity by Research Stage

Stage of the research process	Engagement activities in past research
Topic solicitation, agenda setting and development of research questions	• Provide input on the research topic, prioritization/agenda setting and how to frame the research question^46^–^48^ • Selection of outcomes studied^47^–^49^
Proposal development	• Provide input on lay/plain language summaries for funding applications^48^ • Solicit or amass funding^50^ • Identify and build partnerships with researchers^50^ • Provide support for IRB approval process^50^
Methods/study design	• Select study design^48^,^49^ • Select or develop data collection tools^46^–^48^,^51^
Recruitment	• Recommend strategies for more successful recruitment^48^,^50^
Data collection	• Deliver the research data instrument or conduct participant interviews^47^,^52^ • Develop and host biobanks or registries that serve as sources of data^50^
Data analysis	• Participate in coding the data and data analysis^47^ • Suggest themes for qualitative analysis^48^
Results review, interpretation, and translation	• Interpret research findings^51^ • Highlight most patient-relevant findings^51^ • Identify implications of results for health care delivery^51^,^52^
Dissemination	• Communicate results to other patients, community, and researchers^48^,^51^

IRB Institutional Review Board

Examples were selected from a literature scan of engaged research.^25^ Numbers in parentheses indicate study references

Despite increased attention to engagement, descriptive information about engagement in health research is limited. Some reviews describe engagement in published studies and report impacts of engagement,^1^,^3^,^18^–^21^ but the sparse detail on how engagement is implemented,^22^,^23^ and the lack of systematic characterization and evaluation of engagement^5^,^24^,^25^ highlight the need for additional approaches for obtaining information on engagement. Some earlier studies describe engagement in research in the U.K.,^26^,^27^ but no systematic measures exist to characterize or evaluate engagement. The literature provides insufficient information to permit identification of best practices and guidelines for replication by others. Researchers have acknowledged the need for a better understanding of optimal approaches to engagement, and the opportunity to learn more about engagement from PCORI’s funded projects.^24^ This paper presents our work to collect information about engagement of patients and other stakeholders in PCORI’s first awardees, the 50 PCORI Pilot Projects.

## MATERIALS AND METHODS

### Sample and Data Collection

In 2012, PCORI awarded contracts totaling $31 million for 50 PCORI Pilot Projects (http://www.pcori.org/assets/PCORI-Pilot-Projects-Funding-Announcement-Amendment-1-_v2_-09302011.pdf). The intent of this funding was to advance *methods* for patient-centered outcomes research. Unlike subsequent PCORI funding, these first projects were intended to have an explicit focus on methods (analytic methods, outcome measures, or other specific aspects of research methods). Projects were funded for a maximum of 2 years and $250,000 in direct costs. Engagement of patients and other stakeholders was one criterion by which PCORI evaluated the proposals for funding (as well as significance, investigators, innovation, approach, and environment). PCORI required engagement unless the applicant sufficiently explained why engagement was not pursued.

AcademyHealth, a national health services and policy research organization, was competitively selected to support active management of the PCORI Pilot Projects. PCORI and AcademyHealth collected data regarding engagement of patients and other stakeholders in the design, conduct, and dissemination of the projects (response was voluntary). MaGil IRB provided Institutional Review Board (IRB) approval for secondary analysis of these data.

All 50 PCORI Pilot Project principal investigators (PIs) or their designees were invited to complete an online questionnaire^28^ in July 2013 (approximately 6 to 8 months following project initiation). We sent two reminders 3 and 6 weeks later and a final personalized reminder in early November.

### Measures

The instrument (Appendix A, online) was developed by the PCORI and AcademyHealth team based on PCORI’s conceptual model for patient-centered outcomes research,^29^ key informant interviews with five Pilot Project PIs, qualitative analysis of Pilot Project research proposals, discussion with the PCORI Patient Engagement staff, and literature review, as noted below. In addition to capturing descriptive elements (who, when, and extent of engagement), the instrument content followed the conceptual model categories of *ways to partner, communication, capture and use of perspectives,* and *perceived influence of partners*. To inform future practice, the instrument also addressed challenges to and facilitators of engagement.

#### Stakeholders Engaged

Respondents reported whether they had engaged patients or stakeholders in ways other than as research subjects, the types of stakeholders engaged in their project, and the number of individuals engaged.

#### Level of Engagement

Respondents classified the extent to which each stakeholder type was engaged: 1) Consultation: researchers use stakeholder views to influence decision-making regarding research. Consultation allows researchers to obtain stakeholder views without necessarily being committed to act on them; 2) Collaboration: stakeholders have an ongoing partnership with researchers and greater ownership of the project; or 3) Stakeholder-led: stakeholders initiate, design, and undertake the research process.^6^

#### Nature of Relationships

Respondents reported on the relationship duration (classified as < 1 year, 1 to 3 years, > 3 years); this provided insight into whether the relationship was pre-existing or newly established.

#### Stages of the Research Project

Respondents indicated the stages of research in which each stakeholder type was engaged.^3^,^5^

#### Facilitators of and Challenges for Engagement

Respondents classified a list of possible engagement facilitators on a four-point Likert scale (“Not at all important” to “Critically important”) or “n/a” if not used by their team. Respondents identified challenges to engagement experienced by their team from a list and rated the extent to which challenges were resolved on a three-point Likert scale (“Not at all” to “Completely”). We developed these items based on a review of the literature^5^,^30^–^36^ and knowledge of the investigators’ experiences obtained through interviews and facilitated group discussions with investigators.

#### Evaluation of Stakeholder Influence

Respondents indicated (yes/no) whether they planned to evaluate the influence of stakeholders on the research project.

#### Open-Ended Questions

Respondents answered several open-ended questions about how relationships with stakeholders were established, challenges to engagement, contributions of patients and other stakeholders, and initial learnings about engagement.

### Analysis

Descriptive statistical analyses (including frequencies, proportions) were performed using the survey platform.^28^ The majority of items were created as closed-ended Likert-scaled ratings; we examined percent of sample reporting each response category and interpreted based on response scale. We analyzed responses from open-ended questions (motivations for engagement, establishment of relationships, challenges, lessons learned, and contributions of stakeholder partners) using a descriptive qualitative approach, with each question treated as its own deductive code. Responses to each question were grouped by sub-codes representing themes identified inductively by one member of the team (LEE), and reviewed by another member (LPF). Disagreements were resolved through consensus.

## RESULTS

Researchers from 47 of the 50 projects completed questionnaires (94 %; 33 PIs and 13 other designated research staff). These projects were geographically distributed (Northeast = 30 %, South = 27 %, Midwest = 12 %, West = 30 %). Almost half (46 %) of the projects focused on four health care decision supports; while 30 % focused on research prioritization and analytic methods, 15 % were about developing outcomes instruments, and 13 % related to developing technology and infrastructure for patient-centered outcomes research.

### Engagement of Patients and Other Stakeholders

**Figure 1.**
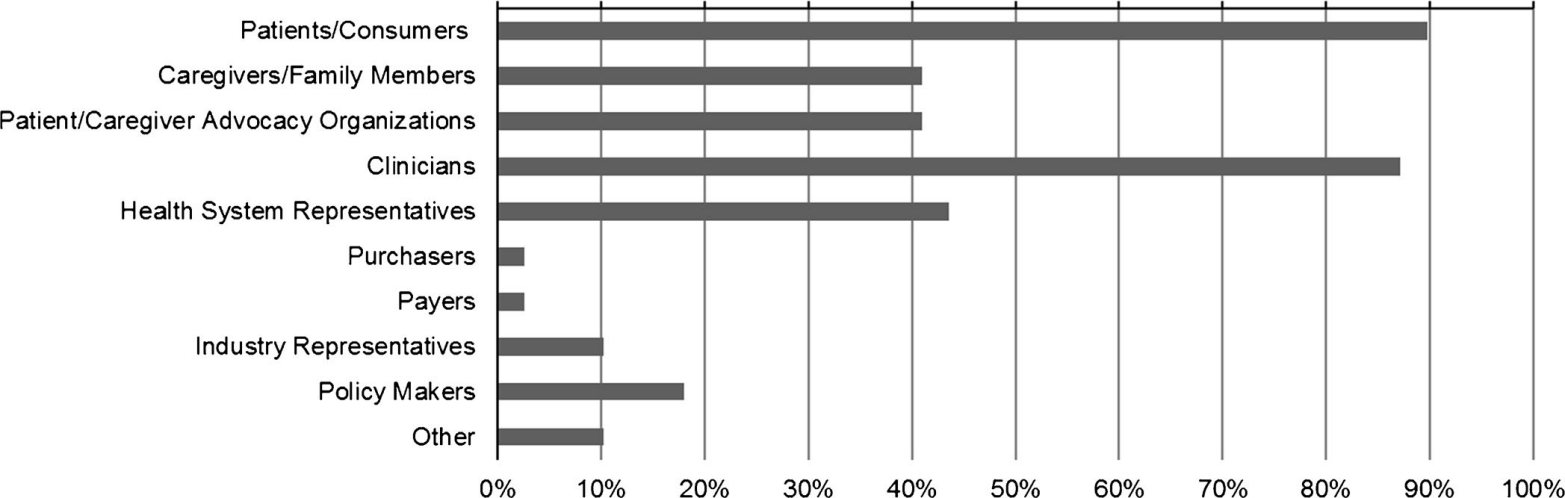
Types of stakeholders engaged in the PCORI pilot projects (among those projects who reported any engagement, *n* = 39).

Most respondents (83 %, *N* = 39) reported engaging patients or other stakeholders in their project in ways other than as research subjects. Among those, investigators most commonly reported engaging patients/consumers (90 %), clinicians (87 %), clinic/hospital or health system representatives (44 %), caregivers/family members (41 %), or patient or caregiver advocacy organizations (41 %) (Fig. 1). Most projects (84 %) engaged at least two types of stakeholder; 22 % reported engaging four or five types of stakeholders.

The primary motivation for engaging patients, their caregivers, and advocacy organizations was to understand their values and perspectives on items such as the impact of a disease, the most appropriate interventions, the importance of specific study outcomes, and research products that meet patients’ needs. The primary motivations for engaging clinicians included their “clinical and methodological expertise” and “insights” into care delivery, obtaining clinician “buy-in,” and ensuring that the research outcomes were clinically meaningful and translatable.

A notable proportion of relationships between researchers and stakeholders existed for < 1 year (37 % of patients, 36 % of caregivers/family members, 19 % of clinicians, 33 % of patient or caregiver advocacy organizations, 27 % of health system representatives). However, some researcher-stakeholder relationships were more long-standing (> 3 years), particularly relationships between researchers and clinicians (50 % of clinicians, 36 % of health system representatives, 27 % of caregivers/family members, 23 % of patients, 22 % of patient or caregiver advocacy organizations). Researchers reported establishing new relationships with patient stakeholders through traditional recruitment in the clinical care setting (e.g., staff outreach, mailings, and flyers); clinician referrals; community outreach; or utilizing investigators’ existing networks with colleagues, patients, and advocacy organizations.

**Figure 2.**
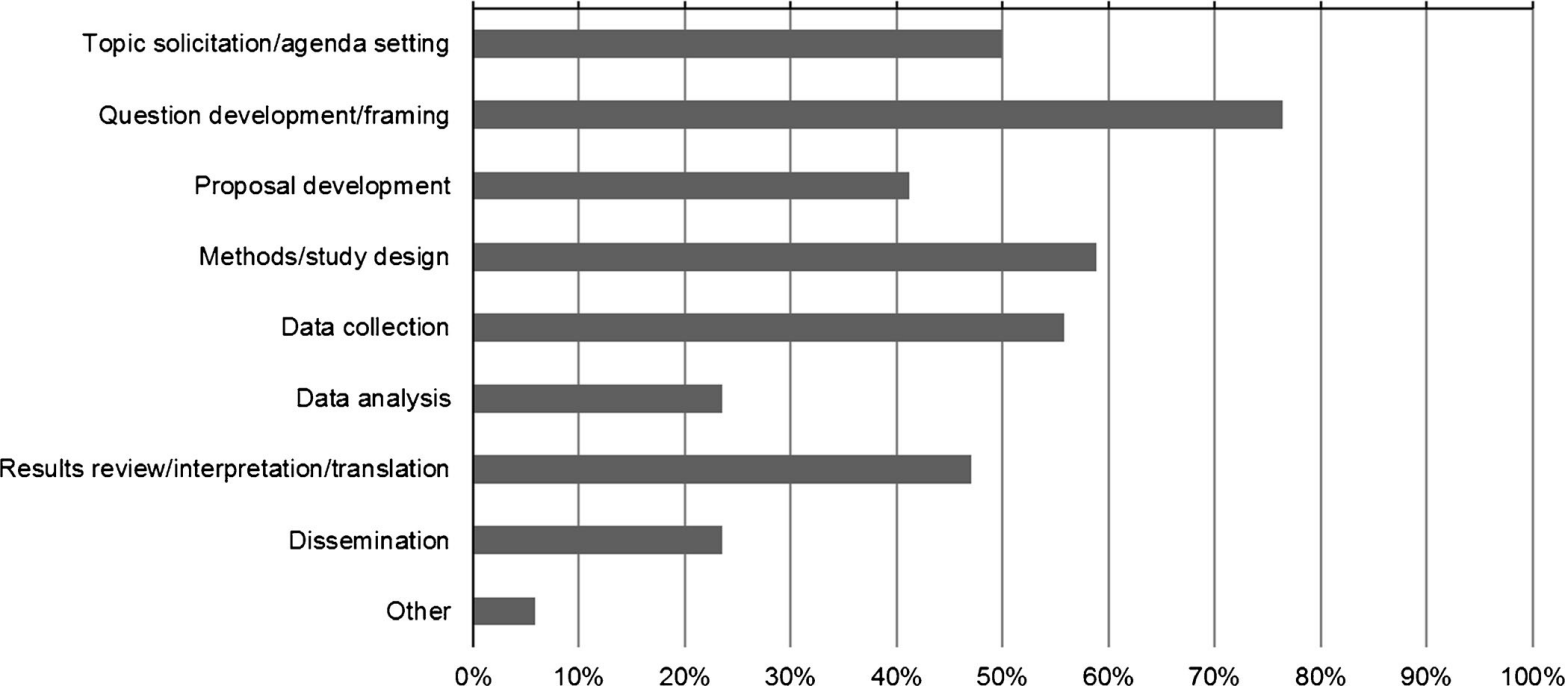
Stages of the research project in which patients were engaged (among PCORI pilot project investigators reporting engagement of patients, *n* = 34).

Regarding engagement of patients specifically, 15 % of projects reported engaging one patient, while 56 % of projects reported engaging ≥ six patients. Most projects reported engaging patients as consultants (35 %) or collaborators (53 %) rather than as co-leaders (6 %). At the time of assessment, patients had engaged most commonly at the stages of research question development, proposal development, study design, data collection, topic solicitation, and results review (Fig. 2). Respondents engaging other stakeholder types gave similar responses regarding the level of engagement and research stages (Appendix B, online).

### Challenges to and Facilitators for Engagement

The most commonly reported challenges to engagement were lack of stakeholder time (46 %), lack of research team time (35 %), lack of stakeholder training/background (30 %), difficulty in finding the appropriate representatives to engage (27 %), lack of research team resources (24 %), and lack of research team training/background (22 %). Respondents generally described these challenges as “partially resolved,” and shared some tangible strategies (Table 2). Respondents also reported establishing trust and learning how to work together as challenges. Some solutions to these challenges included allowing ample opportunity for stakeholders to ask questions, communicating through multiple channels, having face-to-face meetings, making time to socialize, and training researchers on engagement.

**Table 2.** Challenges to Engaging Patients and Other Stakeholders in Research Reported by PCORI Pilot Projects Investigators (*N* = 37)

		Resolved			
Challenge	%	Not at all %	Partially %	Completely %	Strategies for resolution
Lack of stakeholder time	46	6	65	29	• Surveying stakeholders and planning meetings with significant lead time • Hold meetings less frequently or with fewer stakeholders • Provide maximum compensation
Lack of research team time	35	8	77	15	NR
Lack of stakeholder training/background	30	0	73	27	• Seeking out support from existing resources at their institution
Difficulty finding appropriate representatives to engage	27	10	70	20	• Expand their stakeholder recruitment by networking within the group of stakeholders already engaged
Lack of research team resources	24	0	78	22	NR
Lack of research team training/background	22	0	63	38	• Researcher training on cultural sensitivity and community engagement
Lack of stakeholder resources	16	17	83	0	NR
Lack of perceived value among stakeholders	11	25	75	0	NR
Lack of perceived value among research team	8	0	100	0	NR

*NR* none reported

**Figure 3.**
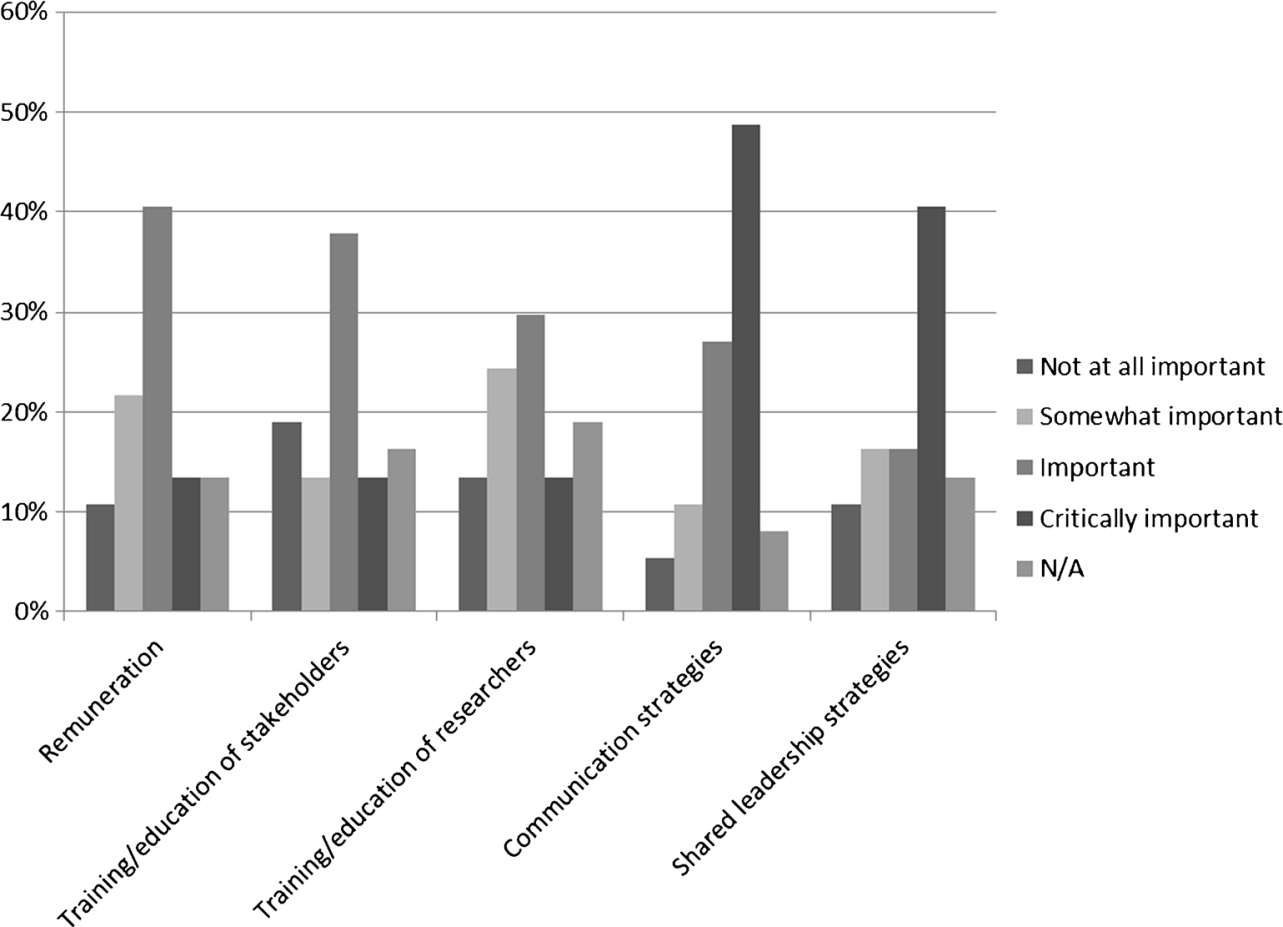
Facilitators of engaging patients and other stakeholders in research (among PCORI pilot projects reporting any engagement, *n* = 36).

Respondents most commonly endorsed communication strategies and shared leadership approaches as “critically important” facilitators (49 and 41 %, respectively). Examples of communication strategies included: trying different arrangements (big group, small group, individual) to see what works best; maintaining regular contact with stakeholders; interacting through multiple channels (online, teleconference, in-person); using non-technical language; and holding pre-meetings with stakeholders to go over items to be discussed in larger meetings. Examples of shared leadership strategies included: adopting a perspective of shared responsibility for decision-making, training of research team members, and involving research partners in research team meetings. Respondents also endorsed remuneration, training of stakeholders, and training of researchers as “important” (41 %, 38 %, and 30 %, respectively) (Fig. 3).

### Early Lessons Learned about Engagement

Early lessons learned (Table 3) suggest the benefits of establishing meaningful partnerships with stakeholders rather than engaging stakeholders merely to fulfill a requirement. Researchers reported the importance of selecting stakeholders strategically to fit project needs, continuously involving stakeholders, adapting to the practical needs of stakeholders, and clearly defining roles and expectations for research partners. Respondents also emphasized the importance of in-person contact for relationship building.

**Table 3.** Summary of Main Themes Identified from Responses to Select Open-Ended Questions

Early Lessons Learned “Based on your interactions with all of these stakeholders up to this point, what initial learnings can you offer to others regarding engaging patients and other stakeholders in research?” (*N* = 35)	
Theme 1: Seek genuine partnership	Respondents emphasized that partnerships with stakeholders must be genuine. For example, one respondent commented, “their participation was enhanced because they quickly realized that their role was not symbolic in nature but was integral to the project’s development in many ways.” Respondents sought to actively involve stakeholders in the decision-making process, which contributed to shared ownership of the project.
Theme 2: Select stakeholders strategically	Respondents recognized the need to strategically select stakeholders representing a variety of different communities or viewpoints and the need to identify the necessary viewpoints to match project specific goals. For example, one respondent described their work as follows: “The three types of stakeholders are quite different and all have been essential to the project. We would not have written a proposal without the administrative stakeholders, the patients and providers are describing the issue of behavioral health within the clinic and identifying their preferences in patient-centered care, and the stakeholder group is deciding how they can feasibly change the way they administer services to meet these needs.”
Theme 3: Continuously involve stakeholders	Respondents acknowledged the need to “start early” and “ask often” as patient and other stakeholders have unique knowledge and improvements to add to the research process along the entire research continuum.
Theme 4: Adapt to the practical needs of stakeholders	Respondents noted the benefits of addressing the practical needs of stakeholders to facilitate their participation (e.g., scheduling meetings outside of traditional working hours, providing transportation).
Theme 5: Define expectations and roles	Respondents identified establishing “parameters and expectations for roles”, giving stakeholders guidance, and allowing time for stakeholders to “get comfortable with their roles” as important tasks.
Theme 6: Use in-person contact to build relationships	To establish strong working relationships, respondents recommended having multiple meetings and underscored the importance of face-to-face contact. While technology and electronic interfaces were noted as facilitators of collaborations across distances, several respondents highlighted the importance of laying the foundation for partnerships through initial in-person meetings.
Contributions of Patients and Other Stakeholders “Up to this point, what are the most significant contribution(s) made by these patients and other stakeholders?” (*N* = 36)	
Theme 1: Changes to project outcomes or goals	Researchers reported that engagement of patients and stakeholders led to a shift in the outcomes of interest to the project. As one respondent described, engaging patients helped the team realize that researchers’ priorities “do not always match priorities of patients.” Another respondent noted “I can say with confidence that our project (the methods and even the project goals) have evolved, in some cases dramatically, based on our collaborations with stakeholders.”
Theme 2: Changes to project methods	Respondents indicated that input through engagement affected their methods in a variety of ways. As one respondent noted, “patient/family collaborators' input was absolutely essential to developing our methods.” For example, patients and stakeholders helped researchers “develop appropriate plans and processes for recruiting patients and interviewing them.” They also helped to improve the ease of data collection, which the respondent indicated may help to speed data collection.
Theme 3: Enhanced access to populations or study settings	Respondents reported that stakeholders facilitated access to clinical professionals and clinical settings in which studies can be conducted.
Theme 4: Modifications to interventions	Stakeholders’ feedback was said to have influenced the development of study interventions, for example, by increasing their ease of use or the manner in which they were implemented.
Theme 5: Refinement of instruments and interview questions	Input into instrument development included pilot testing study instruments, giving “strategic advice” about the length of surveys, and ensuring that the right questions are included for both quantitative and qualitative methods of data collection. As one respondent noted, “our patient collaborators have fundamentally changed the way we ask important questions of patients and families.”
Theme 6: Interpretation and dissemination of results	While most projects were still in the early stages, contributions of patients and stakeholders for interpreting and disseminating results were beginning to emerge. Respondents identified meaningful patient and stakeholder contributions to the interpretation of qualitative findings. One respondent also noted, “they have also changed the way we share study results with kids and families. I mean, they have SERIOUSLY changed things.” Multiple respondents indicated that they expect to engage stakeholders in the interpretation and dissemination of results once findings are available.

### Contributions of Patients and Other Stakeholders

Twenty-one projects (57 %) planned to assess the influence of stakeholders on the research process. The themes that emerged regarding early tangible contributions to the research projects from patients and stakeholders included changes to the project outcomes or goals, changes to the project methods, enhanced access to study populations or study settings, modifications to the study interventions, and refinement of the study instruments or interview questions. Although still in the early stages, several projects identified contributions of patients and stakeholders to results interpretation and dissemination of findings (Table 3).

## DISCUSSION

To address the need for more information about engagement in research, we created and fielded a data collection tool to obtain information about stakeholder engagement practices and strategies employed in PCORI’s Pilot Projects. In the first 6 to 12 months of these projects, researchers most commonly engaged patients, and the majority of projects engaged multiple types of stakeholders. Compared to the published literature, which reflects lesser engagement of other types of stakeholders,^1^,^18^ many PCORI Pilot Projects reported engaging clinicians (> 85 %), and a substantial number of projects reported engaging with caregivers, advocacy organizations, and health system representatives (>40 % for each). However, similar to previous studies, few investigators reported engaging purchasers, payers, policy makers, and the life sciences industry. This pattern may reflect the basic methods research focus of the funding; the stakeholders engaged by these awardees are those most proximal to the methods questions addressed. Subsequent PCORI funding that has a focus on comparative effectiveness research rather than methods research is expected to expand the universe of stakeholders engaged. Our findings highlight the need for a better understanding of how the views from multiple types of stakeholders are incorporated into a study and of the impact of relationship length and quality on the research process. While engagement was most frequently reported in developing research questions, consistent with other reports,^1^,^37^ the extent of engagement in data collection, data analysis, interpretation of results, and dissemination is noteworthy. Engagement in these later phases is expected to increase as projects progress. While the challenges, facilitators, and lessons learned reported here are generally consistent with the existing literature,^3^,^5^,^19^,^20^^,^^38^–^40^ this study is unique in capturing this information systematically and assessing the relative importance across a group of projects. Our findings collectively highlight the effort, attention, resources, and flexibility required for concerted engagement. Across these survey responses, for example, respondents emphasized meaningful and continuous partnerships, shared leadership strategies, communication through multiple strategies that fit specific stakeholder partners, and adaptation to the practical needs of stakeholders. Similarly, others highlighted the importance of sharing power for successful research engagement.^41^ Lack of time—both for stakeholders and researchers—was the most frequently noted challenge and was identified as a top barrier to engagement in a recent survey of patients and clinicians.^39^ Thus, it is critical to recognize resource needs and develop strategies that maximize stakeholder input while minimizing the time burden, and the responses provide support for elements in the conceptual model that helped to inform these items.^29^

Most challenges experienced were only partially resolved. Challenges to engagement remain to be addressed in terms of individual research team process as well as through institutional and funding agency infrastructure. Some issues have tangible solutions (e.g., financial support of partnership in research budgets, matching researchers with stakeholder partners); others require change in social norms (e.g., developing meaningful partnerships). PCORI supports several efforts to address these issues, including funding opportunities for developing research partnerships (http://www.pcori.org/funding-opportunities/pipeline-to-proposal-awards/) and for developing training and building capacity for engagement (http://www.pcori.org/funding-opportunities/eugene-washington-pcori-engagement-awards/).

This study also demonstrates the contributions that engaged stakeholders had already made in the first year of the projects. Researchers reported that patient and stakeholder input resulted in changes to study outcomes, goals, methods, interventions, and materials. These contributions may enhance the appropriateness of study materials,^19^,^21^ and the relevance of the research questions and outcomes^1^,^3^,^18^,^21^,^22^,^42^–^44^ to the study population. Some projects reported stakeholder contributions to the interpretation of qualitative data, which may help researchers to contextualize findings for specific audiences.^3^,^21^ Increased access to study populations or settings suggests that engagement may mitigate commonly encountered challenges with recruitment and retention.^3^,^18^,^22^,^43^ These results are consistent with available evidence on positive impacts of engaged research on research process and dissemination of results.^1^,^6^^,^^18^,^21^,^22^,^42^–^45^ More research is needed to better understand how engagement impacts research quality, relevance, and dissemination.

While the findings represent significant progress towards systematically understanding engagement in research, several limitations should be considered. We obtained only researchers’ perspectives, which may differ from the views of other stakeholders engaged.^26^ This tool did not assess principles of engaged research (e.g., trust, respect) that have been identified as important to the success of engagement. While others have noted important negative impacts of engagement,^21^ the current tool did not specifically ask about these effects and thus did not provide new insights on drawbacks. Obtaining information at later stages of research projects will permit evaluation of engagement outcomes.

Additional information about construct validity of the measure is needed. Results obtained here should be compared to other forms of project reporting, including open-ended project status updates required of awardees and conversations between funding agency staff and awardees. Future data collection efforts should include appropriately timed repetition of key items. Additionally, further cognitive testing of items is warranted to ensure item intent is understood by respondents and that responses reflect respondent intent.

The generalizability of these findings may be limited because respondents were investigators on methods projects rather than health outcomes or CER. Because these respondents were the first PCORI awardees, they also may have more interest in and experience with engagement than the broader health research community. Thus, their knowledge and attitudes about engagement may be more positive. Finally, these self-report data may overestimate research engagement, particularly given the role of the research funder in data collection. Many of these limitations will be addressed in future data collection efforts with PCORI awardees.

Engagement in research is becoming more widespread as the value of end-user needs and perspectives is recognized, and as funding initiatives requiring or encouraging engagement proliferate. Since the PCORI Pilot Projects were funded, researchers have become more familiar with the concept of research engagement. PCORI has provided additional guidance to applicants regarding its funding requirements related to engagement in research and is continuing to collect information on engagement from PIs and their patient and stakeholder partners to inform the literature and PCORI’s policies regarding research engagement.

The evidence on engagement will grow if researchers systematically document more information about engagement and its impact on individual projects in publications,^1^,^23^ if relevant medical subject headings (MESH) indexing terms for patient engagement are developed,^3^ and if information on engagement is systematically collected from researchers and research partners. PCORI continues to collect and use learnings from engagement in funded projects. Future research should evaluate the impact of engagement at each stage of the research process, including the effects of engagement in interpretation of results and dissemination on the clinical uptake of findings. Studies should consider both positive impacts and potential undesirable consequences of engagement, and strategies for mitigating unintended consequences. Furthermore, future evaluations should identify which strategies for engagement—which stakeholders to involve, how to involve them, and at what stages of process—have the greatest positive impact on research quality and usefulness to patients and their caregivers and other end users of the research. Ultimately, evaluations demonstrating links between engagement and longer-term outcomes such as health decision making and health outcomes^29^ are needed to develop evidence-based guidance about the best approaches to engagement in research.

## Electronic supplementary material

ESM 1 (DOCX 25 kb)

ESM 2 (DOCX 54 kb)

## References

[1] Concannon TW, Fuster M, Saunders T, Patel K, Wong JB, Leslie LK (2014). A systematic review of stakeholder engagement in comparative effectiveness and patient-centered outcomes research. J Gen Intern Med.

[2] Deverka PA, Lavallee DC, Desai PJ, Esmail LC, Ramsey SD, Veenstra DL (2012). Stakeholder participation in comparative effectiveness research: defining a framework for effective engagement. J Comp Eff Res.

[3] **Shippee ND, Domecq Garces JP, Prutsky Lopez GJ, Wang Z, Elraiyah TA, Nabhan M, et al.** Patient and service user engagement in research: a systematic review and synthesized framework. Health Expect. 2013. 10.1111/hex.12090 PMC5060820 23731468

[4] Patient-Centered Outcomes Research Institute (PCORI). The PCORI Methodology Report. 2013. Available at: http://www.pcori.org/assets/2013/11/PCORI-Methodology-Report.pdf Accessed June 9, 2015.

[5] Mullins CD, Abdulhalim AM, Lavallee DC (2012). Continuous patient engagement in comparative effectiveness research. JAMA.

[6] Hanley B, Bradburn J, Barnes M, Evans C, Goodare H, Kelson M (2004). Involving the public in NHS public health, and social care research: briefing notes for researchers.

[7] Macaulay AC, Commanda LE, Freeman WL, Gibson N, McCabe ML, Robbins CM (1999). Participatory research maximises community and lay involvement. North American primary care research group. BMJ.

[8] Green LW, Mercer SL (2001). Can public health researchers and agencies reconcile the push from funding bodies and the pull from communities?. Am J Public Health.

[9] Canadian Institute for Health Research (CIHR). Strategy for Patient-Oriented Research Patient Engagement Framework. 2015. Available at: http://www.cihr-irsc.gc.ca/e/documents/spor_framework-en.pdf. Accessed Apr 7, 2015.

[10] **Sarrami Foroushani P, Travaglia J, Eikli M, Braithwaite J.** Consumer and Community Engagement: A review of the literature. 2012. Available at: http://www.aci.health.nsw.gov.au/__data/assets/pdf_file/0010/165592/Consumer-and-community-engagement-literature-review.pdf. Accessed Apr 7, 2015.

[11] **Viswanathan M, Ammerman A, Eng E, Garlehner G, Lohr KN, Griffith D, et al.** Community-based participatory research: assessing the evidence. Evid Rep Technol Assess (Summ). 2004(99):1–8. PMC4780908 15460504

[12] Whitlock EP, Lopez SA, Chang S, Helfand M, Eder M, Floyd N (2010). AHRQ series paper 3: identifying, selecting, and refining topics for comparative effectiveness systematic reviews: AHRQ and the effective health-care program. J Clin Epidemiol.

[13] Food and Drug Administration (FDA). The Voice of the Patient: A Series of Reports from FDA's Patient-Focused Drug Development Initiative. 2014. Available at: http://www.fda.gov/ForIndustry/UserFees/PrescriptionDrugUserFee/ucm368342.htm. Accessed June 9, 2015.

[14] National Heart Lung and Blood Institute (NHLBI). From Public Advocacy to Research Priorities: NHLBI Listens and Responds. 2004. Available at: http://www.nhlbi.nih.gov/files/docs/public/nhlbi-listens.pdf. Accessed June 9, 2015.

[15] National Institutes of Health. Clinical and Translational Science Awards (CTSA): Communities & Research. 2015. Available at: http://www.ncats.nih.gov/ctsa/community. Accessed June 11, 2015.

[16] Patient Protection and Affordable Care Act of 2010, Pub. L. no. 111–148, 124 Stat. 727, Sect. 6301.

[17] Frank L, Basch E, Selby JV (2014). Patient-centered outcomes research I. The PCORI perspective on patient-centered outcomes research. JAMA.

[18] Forsythe LP, Szydlowski V, Murad MH, Ip S, Wang Z, Elraiyah TA (2014). A systematic review of approaches for engaging patients for research on rare diseases. J Gen Intern Med.

[19] Boote J, Baird W, Sutton A (2011). Public involvement in the design and conduct of clinical trials: a narrative review of case examples. Trials.

[20] Boote J, Baird W, Sutton A (2012). Involving the public in systematic reviews: a narrative review of organizational approaches and eight case examples. J Comp Eff Res.

[21] Brett J, Staniszewska S, Mockford C, Herron-Marx S, Hughes J, Tysall C (2014). Mapping the impact of patient and public involvement on health and social care research: a systematic review. Health Expect.

[22] Staley K (2009). Exploring impact: public involvement in NHS, public health, and social care research.

[23] Staniszewska S, Brett J, Mockford C, Barber R (2011). The GRIPP checklist: strengthening the quality of patient and public involvement reporting in research. Int J Technol Assess Health Care.

[24] Workman T, Maurer M, Carman K (2013). Unresolved tensions in consumer engagement in CER: a US research perspective. J Comp Eff Res.

[25] Esmail L, Moore E, Rein A (2015). Evaluating patient and stakeholder engagement in research: moving from theory to practice. J Comp Eff Res.

[26] Barber R, Boote JD, Cooper CL (2007). Involving consumers successfully in NHS research: a national survey. Health Expect.

[27] Hanley B, Truesdale A, King A, Elbourne D, Chalmers I (2001). Involving consumers in designing, conducting, and interpreting randomised controlled trials: questionnaire survey. BMJ.

[28] Qualtrics Software, Version 57764. Provo, UT: Qualtrics Research Suite; 2014.

[29] Frank L, Forsythe L, Ellis L, Schrandt S, Sheridan S, Gerson J (2015). Conceptual and practical foundations of patient engagement in research at the patient-centered outcomes research institute. Qual Life Res.

[30] Mitton C, Smith N, Peacock S, Evoy B, Abelson J (2009). Public participation in health care priority setting: a scoping review. Health Policy.

[31] **Mallery C, Ganachari D, Fernandez J, Smeeding L, Robinson S, Moon M, et al.** Innovative Methods in Stakeholder Engagement: An Environmental Scan. Prepared by the American Institutes for Research under contract No. HHSA 290 2010 0005 C. AHRQ Publication NO. 12-EHC097-EF. Rockville, MD2012.

[32] **McKenzie A, Hanley B.** Consumer and Community Participation in Health and Medical Research: A Practical Guide for Health and Medical Research Organizations. Australia The University of Western Australia and The Telethon Institute for Child Research; 2009.

[33] Minogue V, Girdlestone J (2010). Building capacity for service user and carer involvement in research: the implications and impact of best research for best health. Int J Health Care Qual Assur.

[34] **Lopez MH, Holve E, Rein A, Winkler J.** Involving Patients and Consumers in Research: New Opportunites for Meaningful Engagement in Research and Quality Improvement. EDM Forum, AcademyHealth. June 2012.

[35] Saunders C, Crossing S, Girgis A, Butow P, Penman A (2007). Operationalising a model framework for consumer and community participation in health and medical research. Aust New Zealand Health Policy.

[36] Stacciarini JM, Shattell MM, Coady M, Wiens B (2011). Review: community-based participatory research approach to address mental health in minority populations. Community Ment Health J.

[37] Stewart RJ, Caird J, Oliver K, Oliver S (2011). Patients’ and clinicians’ research priorities. Health Expect.

[38] **Curtis P, Slaughter-Mason S, Thielke A, Gordon C, Pettinari C, Ryan K, et al.** PCORI Expert Interviews Project: Final Report. Portland, OR 2012.

[39] **Forsythe LP, Frank L, Walker KO, Anise A, Wegener N, Weisman H, et al.** Patient and Clinician Views on Comparative Effectiveness Research and Engagement in Research. J Comp Eff Res. in press. 10.2217/cer.14.52 25565066

[40] Oliver S, Clarke-Jones L, Rees R, Milne R, Buchanan P, Gabbay J (2004). Involving consumers in research and development agenda setting for the NHS: developing an evidence-based approach. Health Technol Assess.

[41] Westfall JM, Fagnan LJ, Handley M, Salsberg J, McGinnis P, Zittleman LK (2009). Practice-based research is community engagement. J Am Board Fam Med.

[42] Caron-Flinterman JF, Broerse JE, Bunders JF (2005). The experiential knowledge of patients: a new resource for biomedical research?. Soc Sci Med.

[43] Domecq JP, Prutsky G, Elraiyah T, Wang Z, Nabhan M, Shippee N (2014). Patient engagement in research: a systematic review. BMC Health Serv Res.

[44] **Garces JPD LG, Wang Z, Elraiyah TA, Nabham M, Campana JPB, et al.** Eliciting Patient Perspective in Patient-Centered Outcomes Research: A Meta Narrative Systematic Review. Mayo Clinic, Rochester. 2012. Available at: http://www.pcori.org/assets/Eliciting-Patient-Perspective-in-Patient-Centered-Outcomes-Research-A-Meta-Narrative-Systematic-Review1.pdf. Accessed June 9, 2015.

[45] Nilsen ES, Myrhaug HT, Johansen M, Oliver S, Oxman AD (2006). Methods of consumer involvement in developing healthcare policy and research, clinical practice guidelines and patient information material. Cochrane Database Syst Rev.

[46] Staniszewska S, Jones N, Newburn M, Marshall S (2007). User involvement in the development of a research bid: barriers, enablers and impacts. Health Expect.

[47] Lophatananon A, Tyndale-Biscoe S, Malcolm E, Rippon HJ, Holmes K, Firkins LA (2011). The James Lind alliance approach to priority setting for prostate cancer research: an integrative methodology based on patient and clinician participation. BJU Int.

[48] Lindenmeyer A, Hearnshaw H, Sturt J, Ormerod R, Aitchison G (2007). Assessment of the benefits of user involvement in health research from the Warwick diabetes care research user group: a qualitative case study. Health Expect.

[49] Edwards V, Wyatt K, Logan S, Britten N (2011). Consulting parents about the design of a randomized controlled trial of osteopathy for children with cerebral palsy. Health Expect.

[50] Terry SF, Terry PF, Rauen KA, Uitto J, Bercovitch LG (2007). Advocacy groups as research organizations: the PXE international example. Nat Rev Genet.

[51] Barber R, Beresford P, Boote J, Cooper C, Faulkner A (2011). Evaluating the impact of service user involvement on research: a prospective case study. Int J Consum Stud.

[52] Fern LA, Taylor RM, Whelan J, Pearce S, Grew T, Brooman K (2013). The art of age-appropriate care: reflecting on a conceptual model of the cancer experience for teenagers and young adults. Cancer Nurs.

