# Supplementary material for: Patient and Stakeholder Engagement in the PCORI Pilot Projects: Description and Lessons Learned
Source: J Gen Intern Med. 2015 Jul 10;31(1):13–21. doi: 10.1007/s11606-015-3450-z (PMC4700002; doi:10.1007/s11606-015-3450-z)
Supplement: Supplementary file 1 — (DOCX 25 kb) [file 11606_2015_3450_MOESM1_ESM.docx]

**Appendix A: Data collection tool**

1. At this point in your PCORI research project, have you engaged patients or other stakeholders in your project in ways *other* than as research subjects?
   - Yes
   - No *(skip to Q 24)*
2. Which of the following stakeholder communities have you engaged in your PCORI project in ways *other* than as research subjects? Please select all that apply, but only one category per stakeholder engaged (i.e., please select the community with which a given stakeholder would most closely identify for the purposes of your project).

- Patient/Consumer (unaffiliated individual)
- Caregiver/Family Member of Patient (unaffiliated individual)
- Patient/Consumer/Caregiver Advocacy Organization
- Clinician (e.g. Nurse, Physician, etc., or an organization that represents clinicians)
- Clinic/Hospital/Health System Representative (e.g. FQHC, Rural Health Clinic, etc., or an organization that represents hospitals/health systems)
- Purchaser (e.g. Employer or an organization that represents purchasers)
- Payer (e.g. Health Insurer, Medicaid, etc., or an organization that represents payers)
- Industry Representative (e.g. Device or Pharmaceutical Manufacturer, or an organization that represents industry)
- Policy Maker (e.g. State Legislator, Executive Agency Employee, etc., or an organization that represents policy makers)
- Other (Please describe)

1. For each type of stakeholder engaged in your project, please indicate the nature of their involvement. Check all that apply.

|  | Stakeholder-led^^[[1]](#footnote-1)^^ | Collaborator^^[[2]](#footnote-2)^^ | Consultant^^[[3]](#footnote-3)^^ | Other |
| --- | --- | --- | --- | --- |
| <insert stakeholder> |  |  |  |  |
| <insert stakeholder> |  |  |  |  |
| <insert stakeholder> |  |  |  |  |

*For each type of stakeholder selected, the following questions will be presented (the term “patient/consumer (unaffiliated individual)” is used as an example below, but other stakeholder types might include those specified in Q 2 above). The full set of questions (4 thru 11) will be asked for each selected stakeholder type.*

1. How many <insert stakeholder> have you engaged in your project thus far?
   - 1
   - 2
   - 3
   - 4
   - 5
   - More than 5
2. How was the relationship with this/these <insert stakeholder> established?
3. For how long have you been working with this this/these <insert stakeholder> (on this or other research projects)?
4. What challenges did you encounter in establishing the relationship with this/these <insert stakeholder>?
5. Please describe any efforts made by your team to overcome these challenges.
6. What was your primary motivation for engaging this/these <insert stakeholder> in your research project?
7. Up to this point, in what stage(s) in the research process have you worked with this/these <insert stakeholder>? Select all that apply.
   - topic solicitation/agenda setting
   - question development/framing
   - proposal development
   - methods/study design
   - data collection
   - data analysis
   - results review/interpretation/translation
   - dissemination
   - other (please specify)
8. Up to this point, how often have you or other members of your research team emailed, spoken to, or met with (in person) this/these <insert stakeholder> about the project?
   - not at all
   - fewer than 4 times a year
   - once every 2-3 months
   - approximately once a month
   - 2-3 times a month
   - more than 3 times a month

*NOTE: Repeat Qs 4 – 11 with each stakeholder type specified by the respondent in Q 2; then proceed to Q 12.*

1. Based on your interactions with all of these stakeholders up to this point, what initial learnings can you offer to others regarding engaging patients and other stakeholders in research?
2. Up to this point, what are the most significant contribution(s) made by these patients and other stakeholders?

Facilitators

1. Please rate the importance of each of the following facilitators of engagement of patients or other stakeholders in research for your project. If no facilitators were used by your team, please select the “n/a” option corresponding to “none.”

|  | Not at all Important | Somewhat important | important | Critically important | NA |
| --- | --- | --- | --- | --- | --- |
| Remuneration (e.g., honoraria, travel support) |  |  |  |  |  |
| Training/education of stakeholders |  |  |  |  |  |
| Training/education of researchers |  |  |  |  |  |
| Communications processes / tools |  |  |  |  |  |
| Shared leadership strategies |  |  |  |  |  |
| Other 1 |  |  |  |  |  |
| Other 2 |  |  |  |  |  |
| None |  |  |  |  |  |

1. Please describe the “other” facilitators your research team used to facilitate engagement of patients or other stakeholders.

Challenges

1. Up to this point, which of the following challenges have members of your team experienced in engaging these patients or other stakeholders? Please select all that apply.
   - lack of research team training/background
   - lack of stakeholder training/background
   - lack of research team time, lack of stakeholder time
   - lack of research team resources, lack of stakeholder resources
   - lack of perceived value among research team
   - lack of perceived value among stakeholders
   - difficulty in finding the appropriate representatives to engage
   - other, please describe:
   - none (skip to Q 19)
2. To what extent have you been able to resolve these challenges? See below.

|  | Not at all resolved | Partially resolved | Completely resolved |
| --- | --- | --- | --- |
| lack of research team training/background in engagement of patients and other stakeholders |  |  |  |
| lack of stakeholder training/background in research process / methods |  |  |  |
| Lack of research team time |  |  |  |
| Lack of stakeholder time |  |  |  |
| Lack of research team resources |  |  |  |
| Lack of stakeholder resources |  |  |  |
| Lack of perceived value among research team |  |  |  |
| Lack of perceived value among stakeholders |  |  |  |
| difficulty in finding the appropriate representatives |  |  |  |
| Other 1 |  |  |  |
| Other 2 |  |  |  |

1. Please describe how you resolved these challenges.
2. Do you plan to assess the level of influence of these patients and other stakeholders on the research project?
   - Yes, please describe: *(Skip to Q 20)*
   - No
3. Would you be interested in assessing the level of influence of these patients and/or stakeholders on your research project?
4. Up to this point, have you collected any information about the experiences of patients and other stakeholders engaged in this research project (e.g., level of satisfaction with their role)?
   - Yes
   - No *(skip to Q 23)*
5. Please describe the type of information you have collected regarding the experiences of these patients and other stakeholders.
6. Do you have plans to collect information on the experiences of patient and other stakeholders engaged in this project in the future?
   - Yes
   - No
7. Please feel free to share any further comments or suggestions for PCORI on engaging patients and other stakeholder engagement.

1. Stakeholder-led research: stakeholder(s) design and undertake the research and researchers are invited to participate at the invitation of the stakeholder(s). [↑](#footnote-ref-1)
2. Collaborators have an ongoing partnership/affiliation with the researchers and healthcare professionals in the research process, and are formally engaged to complete a specific research project, and therefore have greater ownership of the project (e.g., steering committee, co-investigator). [↑](#footnote-ref-2)
3. Consultants are asked about their views on various aspects of the research and researchers use their views to influence decision-making regarding research (e.g., focus groups, interviews, requests for input). Consultation allows the researcher to obtain views’ without necessarily being committed to act on them. [↑](#footnote-ref-3)
