# Supplementary material for: Patient and Stakeholder Engagement in the PCORI Pilot Projects: Description and Lessons Learned
Source: J Gen Intern Med. 2015 Jul 10;31(1):13–21. doi: 10.1007/s11606-015-3450-z (PMC4700002; doi:10.1007/s11606-015-3450-z)
Supplement: Supplementary file 2 — (DOCX 54 kb) [file 11606_2015_3450_MOESM2_ESM.docx]

**Appendix B: Supplemental Results**

*Stages of the Research Project in which Caregivers Were Engaged (among those projects engaging caregivers, n=15)*

*Stages of the Research Project in which Advocacy Organizations were Engaged (among those projects engaging advocacy organizations, n=15)*

*Stages of the Research Project in which Clinicians were Engaged (among those projects engaging clinicians, n=32)*

*Stages of the Research Project in which Hospital or Health System Representatives were Engaged (among those projects engaging hospital or health system representatives, n=16)*
